# Supplementary material for: D-LL-31 enhances biofilm-eradicating effect of currently used antibiotics for chronic rhinosinusitis and its immunomodulatory activity on human lung epithelial cells
Source: PLoS One. 2020 Dec 16;15(12):e0243315. doi: 10.1371/journal.pone.0243315 (PMC7743948; doi:10.1371/journal.pone.0243315)
Supplement: S1 Table — Bacterial suspensions were incubated with antibiotics for 24 h and the results were interpreted according to BD BBL™ Sensi-Disc™ antimicrobial susceptibility test discs. (DOCX) [file pone.0243315.s001.docx]

**S1 Table. Antimicrobial susceptibility of reference strain and representative bacteria from CRS patients.**

| **Bacterial strains** | **Antibiotics** | | | | | | | | | |
| --- | --- | --- | --- | --- | --- | --- | --- | --- | --- | --- |
|  | **AMP** | **CRO** | **CHL** | **ERY** | **GEN** | **KAN** | **OXA** | **PEN** | **TET** | **TOB** |
| *K. pneumoniae* | R | S | S | ND | I | S | ND | ND | I | S |
| *S. epidermidis* | R | S | S | R | S | S | S | S | S | S |
| *P. aeruginosa* | ND | R | R | ND | I | ND | ND | ND | R | S |
| *P. aeruginosa* ATCC27853 | ND | S | S | ND | S | ND | ND | ND | S | S |

ND, Not determined; S, Susceptible; I, Intermediate; R, Resistant.

AMP, Ampicillin; CRO, Ceftriaxone; CHL, Chloramphenicol; ERY, Erythromycin; GEN, Gentamicin; KAN, Kanamycin; OXA, Oxacillin; PEN, Penicillin; TET, Tetracycline; TOB, Tobramycin.
