# Supplementary material for: D-LL-31 enhances biofilm-eradicating effect of currently used antibiotics for chronic rhinosinusitis and its immunomodulatory activity on human lung epithelial cells
Source: PLoS One. 2020 Dec 16;15(12):e0243315. doi: 10.1371/journal.pone.0243315 (PMC7743948; doi:10.1371/journal.pone.0243315)
Supplement: Attachment — Submitted filename: Response to Reviewers.docx [file pone.0243315.s002.docx]

Sakawrat Kanthawong, Ph.D.

Department of Microbiology,

Faculty of Medicine,

Khon Kaen University,

Khon Kaen, THAILAND 40002

Tel./Fax. 66 43 363808

Email: sakawrat@kku.ac.th

October 29, 2020

**Dear Y. Peter Di, Ph.D.**

**Academic Editor**

**PLOS ONE**

**Subject**: Manuscript submission after revised in response to the reviewers’ criticisms (manuscript PONE-D-20-18804 entitled “D-LL-31 enhances biofilm-eradicating effect of currently used antibiotics for chronic rhinosinusitis and its immunomodulatory activity on human lung epithelial cells”)

We would like to thank you for your e-mail dated September 14, 2020, together with the reviewer’s comments. We would like to thank the reviewers for their thoughtful review of the manuscript. They raise important issues and their inputs are very helpful for improving the manuscript. We have carried out the experiments that the reviewers suggested to demonstrate the antimicrobial activity of antibiotic (AMX and TOB) and AMPs (LL-37, LL-31 and their D-form) against *P. aeruginosa* ATCC27853 (bacterial reference strain) in order to compare the activity of D-LL-31 with other AMPs (Fig 2D), have added S1 Table of the Antimicrobial susceptibility of reference strain and representative bacteria from CRS patients and revised Fig 6 showing the cytotoxicity of D-LL-31 (0.05-100 µM) on human lung epithelial cells. We also have revised our manuscript accordingly. The modified parts of the manuscript are highlighted. Please find attached a point-by-point response to reviewer’s concerns.

**‘Journal Requirements:**

**When submitting your revision, we need you to address these additional requirements.**

**1. Please ensure that your manuscript meets PLOS ONE's style requirements, including those for file naming. The PLOS ONE style templates can be found at**

**https://journals.plos.org/plosone/s/file?id=wjVg/PLOSOne_formatting_sample_main_body.pdf and**

**https://journals.plos.org/plosone/s/file?id=ba62/PLOSOne_formatting_sample_title_authors_affiliations.pdf**

**Response:** We have formatted the manuscript accordingly.

**Reviewer #1:**

**Dr. Kanthawong continues her thorough characterization of antimicrobial effects of D-LL-31 in clinically relevant biofilm bacterial infections of mucosal surfaces, here chronic rhinosinusitis.**

**Experiments are carefully conducted and clearly presented. Synergy with antibiotics is demonstrated.**

**Dr. Kanthawong might consider commenting on anti-viral potential of D-LL-31 and provide references to support this direction for translational investigations. See Yu et al https://doi.org/10.1016/j.isci.2020.100999**

**Response:** We thank the reviewer for this suggestion. As suggested by the reviewer, we have added this work in the original manuscript (page 3 line 69).

**Reviewer #2:**

**In this study, Wongkaewkhiaw et al. demonstrated that D-LL-31, the d-enantiomer of LL31 showed significant increase of antimicrobial activities against three pathogens that are associated with chronic rhinosinusitis (CRS). D-LL-31 also showed synergistic effects when combined with clinically used antibiotics. The study design was good in testing the main hypothesis. It was commendable that the authors adopted many relevant methods for the microbiology studies, such as SEM, MIC, MBC, and TSP pegged lids for biofilm studies. Given the urgent need of new antibiotics, I am willing to endorse the good contribution to the field of antimicrobial peptides. However, there are some concerns need to be addressed.**

**1. There were a lot of experimental details missing from the manuscript and the authors need to provide the required information and clarify the experimental details so the data can be evaluated and reproduced by other researchers. For instance, the AMX or TOB concentrations used in synergistic effect (Table 5) and biofilm images (Figure 4) were not presented.**

**Response:** We appreciate the reviewer’s perspective. We did not clearly explain this point in the original manuscript. For synergistic study, we used the IC_50_ and also lower concentrations down to 1/16 of their IC_50_ values of each agent (antibiotics and D-LL-31). We have added the paragraph about the used concentrations of AMX or TOB in Materials and Methods section for clarity (page 10; lines 230-232).

“Briefly, 24 h biofilms on TSP pin lids were mixed with the antibiotics (AMX or TOB) and D-LL-31 diluted in 1 mM PPB to final concentrations of IC_50_ down to 1/16 of the IC_50_ values of each agent and incubated at 37°C for 24 h (Table 4).”

Moreover, we have added the used concentration of each treatment in Fig 4 and in the anti-biofilm matrix study paragraph in Materials and Methods section (page 11; lines 244-253).

“After growing the biofilm, D-LL-31 or AMX alone (IC_50_ values; 0.6 µM D-LL-31 and 7925 µM CAZ (2896 µg/ml)) and a combination of both (based on the synergy data; 0.07 µM D-LL-31 and 495 µM CAZ (181 µg/ml)) were used in *K. pneumoniae* biofilm matrix challenge. For *S. epidermidis* biofilm matrix, IC_50_ values of D-LL-31 (0.5 µM) or AMX (33092 µM AMX (12092 µg/ml) and combination of D-LL-31/AMX (0.03 µM D-LL-31 and 2068 µM CAZ (756 µg/ml)) were used. For *P. aeruginosa* biofilm matrix, IC_50_ values of D-LL-31 (4 µM) or AMX (45163 µM (16502 µg/ml)) or TOB (4 µM (2 µg/ml) and combination of D-LL-31/AMX (0.25 µM D-LL-31 and 2823 µM CAZ (1031 µg/ml)) or D-LL-31/TOB (0.5 µM D-LL-31 and 0.2 µM TOB (0.1 µg/ml)) were used. All biofilms were treated at 37°C for 24 h.”

**2. The bacterial strains were isolated directly from patients, which is highly relevant to the study of D-LL-31 within clinical conditions. However, it was not clear how many isolates were used in the subsequent experiments. Did the authors use only one isolate or multiple isolates (from which patients) per bacterial strain for the experiments? Were the antibiogram for the used isolates available and can be provided (even as a supplemental data)? Were the used bacterial isolate(s) representative to the respective bacterial strains? Can the author tried to include some standard laboratory strains such as PAO1 or PA14 for *P. aeruginosa* so that other researchers in this field can easily compare the activity of D-LL-31 to other AMPs.**

**Response:** Three bacterial stains (*P. aeruginosa*, *S. epidermidis* and *K. pneumoniae*) were selected based on the evidence of the biofilm positive under SEM in our study and also showed a high prevalence in previous studies with CRS patients (Zhang et all, 2015; Dlugaszewska et all, 2016 and Karunasagar et al, 2018). Only one isolate of each bacterial strain was selected as representative bacteria in this study. *P. aeruginosa*, *S. epidermidis* and *K. pneumoniae* from patients no. 1, 3 and 5, respectively (Table 2) were used in all experiments. We have added a sentence to explain this in Materials and Methods section (page 7; line 157).

“Three clinical bacterial isolates*, Klebsiella pneumoniae*, *Pseudomonas aeruginosa* and *Staphylococcus epidermidis*, were selected from patients no. 1, 3 and 5, respectively (Table 2) according to the predominant bacterial species that commonly found in clinical studies on CRS”

Moreover, we have now added S1 Table to demonstrate the antibiogram of reference bacterial strain (*Pseudomonas aeruginosa* ATCC27853) and the three used bacterial isolates. Antibiotics susceptibility testing was performed using BD BBL™ Sensi-Disc™ antimicrobial susceptibility test discs (Becton, Dickinson and Company, Franklin Lakes, NJ) and the results were interpreted according to the manufacturer's instructions.

We have added this in Materials and Methods section (page 7; lines 160-161).

“*P. aeruginosa* ATCC27853 was also included in this study as reference strain. Antimicrobial susceptibility test results of the bacterial isolates are shown in S1 Table.”

As suggested by the reviewer, we have performed an experiment to demonstrate the antimicrobial activity of antibiotic (AMX and TOB) and AMPs (LL-37, LL-31 and their D-form) against *P. aeruginosa* ATCC27853 (bacterial reference strain) in order to compare the activity of D-LL-31 with other AMPs (Fig 2D).

We have therefore revised Fig 2 and the paragraph in the results (page 17; lines 359-363).

“TOB at concentrations of 1 and 5 µM showed ≥ 92% killing effect against the clinical isolate *P. aeruginosa* at 1-2 h, but 10 µM was required to kill all bacterial cells (Fig 2C) while only 1 µM TOB showed ≥ 98% killing effect against the reference strain *P. aeruginosa* ATCC2853 at 1 h and both 5 and 10 µM killed all bacterial cells within 1 h (Fig 2D).”

**S1 Table. Antimicrobial susceptibility of reference strain and representative bacteria from CRS patients.**

| **Bacterial strains** | **Antibiotics** | | | | | | | | | |
| --- | --- | --- | --- | --- | --- | --- | --- | --- | --- | --- |
|  | **AMP** | **CRO** | **CHL** | **ERY** | **GEN** | **KAN** | **OXA** | **PEN** | **TET** | **TOB** |
| *K. pneumoniae* | R | S | S | ND | I | S | ND | ND | I | S |
| *S. epidermidis* | R | S | S | R | S | S | S | S | S | S |
| *P. aeruginosa* | ND | R | R | ND | I | ND | ND | ND | R | S |
| *P. aeruginosa* ATCC27853 | ND | S | S | ND | S | ND | ND | ND | S | S |

ND, Not determined; S, Susceptible; I, Intermediate; R, Resistant.

AMP, Ampicillin; CRO, Ceftriaxone; CHL, Chloramphenicol; ERY, Erythromycin; GEN, Gentamicin; KAN, Kanamycin; OXA, Oxacillin; PEN, Penicillin; TET, Tetracycline; TOB, Tobramycin.

**Fig 2. Antimicrobial activity of antibiotics and AMPs against planktonic culture.** Bacterial suspensions of (A) *K. pneumoniae*, (B) *S. epidermidis* and (C) *P. aeruginosa* (D) *P. aeruginosa* ATCC 27853 were incubated with 1, 5 and 10 µM of each peptide for 1 and 2 h. The results are presented as mean values ± SD of three independent experiments, carried out in duplicate.

**3. The biofilm assay description should be clarified regarding the starting bacterial numbers. What were the bacterial numbers used for the biofilm assay? The number seemed to be extremely high for using 200 ul of OD_600_=0.9 bacterial in a 96-well plate well. In addition, the bacterial numbers would also be very different with regards to the three different bacterial strains among PA, KP and SE. Thus, it might not be appropriate to directly compare their biofilm forming ability among different strains as presented in the manuscript.**

**Response:** As suggested by the reviewer, we have repeated the biofilm crystal violet staining assay of all tested bacteria using 10^8^ CFU/ml of bacterial cells instead of OD_600_=0.9 of bacterial suspension. We have added the number of bacterial cells in Materials and Methods section (page 9; line 197).

“Two hundred microlitres of bacterial suspension (10^8^ CFU/ml) were added into a sterile 96-well flat-bottom plate (Nunclon^™^) and the plate was incubated at 37˚C for 3 h. Cell-free medium was used as a control.”

In addition, we have revised the results accordingly (page 18; lines 378-379).

“*P. aeruginosa* showed the greatest tendency for biofilm formation [OD_620_ (mean ± SD); 0.376 ± 0.1], followed by *S. epidermidis* and *K. pneumoniae,* 0.273 ± 0.05 and 0.203 ± 0.04, respectively.”

**4. OD readings in MTT assay has been known to not normally being linear to the total cell numbers because of the different growth condition and proliferation rate. A standard curve between OD readings and cell numbers need to be first established before the OD readings can be used for the calculation of cytotoxicity. The authors need to be aware of this inappropriate use of MTT assay.**

**Response:** As suggested by the reviewer, we have repeated our MTT assay. Firstly, the viability of A549 cells (100-50,000 cells/well) was determined as previously described with some modification (Young et all, 2005) and used for calculation of the cytotoxicity of D-LL-31. We have therefore revised the paragraph in Materials and Methods section (page 12; lines 279-280).

“Briefly, A549 cells were maintained in RPMI-1640 (HyClone^™^, Marlborough, MA) with 10% fetal bovine serum (FBS) (HyClone^™^) and sub-cultured by standard methods using trypsin/EDTA (0.25% trypsin, 0.1% EDTA) (Corning^™^, Corning, NY). A standard curve of A549 cells (100-50,000 cells/well) was constructed as previously described with some modification [43]. For MTT assay, cells were plated at a density of 2.5×10^4^ cells/well in 96-well plates and grown overnight.”

43. Young FM, Phungtamdet W, Sanderson BJ. 2005. Modification of MTT assay conditions to examine the cytotoxic effects of amitraz on the human lymphoblastoid cell line, WIL2NS. Toxicol In Vitro 19:1051-9. https://doi.org/10.1016/j.tiv.2005.05.001 PMID: 16125362.

**5. In addition, the cytotoxicity assay for D-LL-31 was also incorrectly performed. The toxicity data should include higher concentration up to 100 µM similar to what were used in Fig 3. Furthermore, the toxicity data needs to be factored into the calculation of cytokine inhibition as there was minor cytotoxicity at 10 uM, the concentration used in the cytokine study.**

**Response:** With all due respect to the reviewer, the concentrations of D-LL-31 for the cytotoxicity assay ranking from 0.05-10 µM were selected based on the IC_70_ values of D-LL-31 alone and in combination with antibiotics against bacterial isolated from CRS patients under biofilm-stimulating condition (0.8-8.7 µM D-LL-31) and also 10 µM for cytokine antibody arrays. However, we have repeated the cytotoxicity assay for D-LL-31 at concentration ranking from 0.05-100 µM to A549 cells as suggested by the reviewer. We found that treatment with 25 µM D-LL-31 still resulted in more than 80% viable A549 cells while 50-100 µM D-LL-31 resulted in more than 90% cytotoxicity of A549 cells (Fig 6). Generally, the action mechanism of antimicrobial peptides is membrane disruption mediated by electrostatic forces and hydrophobic interaction (Ebenhan et al., 2014); therefore, cytotoxicity to human cells may be observed when high concentration of potential antimicrobial peptides is used. Thus, we have decided to used D-LL-31 combined with antibiotics in order to reduce the cytotoxicity and immunomodulatory effect of peptide in this study.

We have revised Fig 6 and the paragraph in Result section (page 23; lines 481-486).

“More than 90% of cells remained viable after treatment with 12.5 μM D-LL-31, and more than 80% after treatment with 25 μM, indicating that D-LL-31 exhibited low cytotoxicity against A549 cells at concentrations that are effective against the bacteria grown as biofilm (IC_90_ values of D-LL-31 at concentration ranging from 1 to 25 µM). However, treatment with ≥ 50 µM D-LL-31 resulted in more than 90% cytotoxicity of A549 cells.”

**Fig 6. Cytotoxicity of D-LL-31 on human lung epithelial cells.** The cells were incubated with different concentrations of D-LL-31 (0.05-100 µM) for 24 h. The cell viability was assessed using the MTT assay. Data are presented as the mean ± SD (**P*<0.001).

Reference

Ebenhan T, Gheysens O, Kruger HG, Zeevaart JR, Sathekge MM. Antimicrobial peptides: their role as infection-selective tracers for molecular imaging. Biomed Res Int. 2014;2014:867381. doi: 10.1155/2014/867381. Epub 2014 Aug 27. PMID: 25243191; PMCID: PMC4163393.

**6. For figure 1 and table 2, the N of each strains of bacteria should be provided so the data can be interpreted correctly. It is a concern that the sample size may be too small and the results could potentially be biased.**

**Response:** As suggested by the reviewer, we have added a column to present the total number of isolate in each sample (Table 2). As indicated above we have selected one isolate for each bacterial strain.

Moreover, the sample size of this study was 19 patients which using the formula below (Kamangar et al., 2013) resulted in 95% confidence level. The estimated prevalence was 72.67% ± 11.02 (based on the prevalence of biofilm on sinus mucosa from previous studies; Sanclement et al., 2005: Sanderson et al., 2006 and Xiong et al., 2010), with 5% dropout rate.

N = (Z_1-α/2_)^2^ σ^2^

d^2^

N = 1.96^2^× 11.02^2^

5^2^

= 18.66 ~ 19 samples

Reference

Kamangar F, Islami F. Sample size calculation for epidemiologic studies: principles and methods. Arch Iran Med. 2013 May;16(5):295-300. PMID: 23641744.

Sanclement JA, Webster P, Thomas J, Ramadan HH. Bacterial biofilms in surgical specimens of patients with chronic rhinosinusitis. Laryngoscope. 2005 Apr;115(4):578-82. doi: 10.1097/01.mlg.0000161346.30752.18. PMID: 15805862.

Sanderson AR, Leid JG, Hunsaker D. Bacterial biofilms on the sinus mucosa of human subjects with chronic rhinosinusitis. Laryngoscope. 2006 Jul;116(7):1121-6. doi: 10.1097/01.mlg.0000221954.05467.54. PMID: 16826045.

Xiong GY, Chen HH, Wang SQ, Xie XX, Lin W, Shen Q. [Relationship between bacterial biofilms and chronic rhinosinusitis by scanning electron microscopic study]. Zhonghua Er Bi Yan Hou Tou Jing Wai Ke Za Zhi. 2010 Jan;45(1):19-23. Chinese. PMID: 20398532.

**7. Fig 2. As many previous studies have suggested that antimicrobial peptides usually kill bacteria upon contact and the killing is often instant. I wonder why the author picked 1- and 2-hour time points instead of a 1, 5, 10 minutes etc. time frame so that the advantage of peptide instant killing could be showcased. In contrast, AMX and TOB, although effective occasionally, likely wouldn’t work as fast as AMPs such as D-LL-31. The bacterial amount (10^5^) used for Figure 2 was less than the standard required numbers, which may affect the correct interpretation of the peptide potency.**

**Response:** With all due respect to the reviewer, we have selected 1 and 2 h of incubation time for screening and comparing the efficiency of our antimicrobial peptides against various planktonic bacteria with our previous study (Wongkaewkiaw et al., 2019; Kanthawong et al., 2012). Moreover, the number of bacterial cells was prepared approximately 10^5^ CFU/ml (ranging from 3-5 x 10^5^ CFU/ml) according to Broth Microdilution Method from the Clinical and Laboratory Standards Institute (CLSI) protocol which requires a standardized inoculum density of 5 x 10^5^ CFU/ml with an allowable range of 2-8 x 10^5^ CFU/ml. We believe that we did not clearly explain this point in the original manuscript. We have added the exact number of bacterial cells in Materials and Methods section accordingly (page 8; lines 175 and 185).

“Bacterial suspensions (5 x 10^5^ CFU/ml) were added to the microtiter plates, which were then incubated at 37˚C for 24 h.”

“Each bacterial suspension (5 x 10^5^ CFU/ml) in 1 mM potassium phosphate buffer (PPB) (pH 7.0) was added to an equal volume of each antibiotic (AMX and TOB) and AMP (LL-37, LL-31, D-LL-37 and D-LL-31) to reach final concentrations of 1, 5 and 10 µM and incubated at 37 °C for 1 and 2 h.”

Reference

Wongkaewkhiaw S, Taweechaisupapong S, Anutrakunchai C, Nazmi K, Bolscher JGM, Wongratanacheewin S, Kanthawong S. D-LL-31 in combination with ceftazidime synergistically enhances bactericidal activity and biofilm destruction in *Burkholderia pseudomallei*. Biofouling. 2019 May;35(5):573-584. doi: 10.1080/08927014.2019. 1632835. Epub 2019 Jul 8. PMID: 31282211.

Kanthawong S, Bolscher JG, Veerman EC, van Marle J, de Soet HJ, Nazmi K, Wongratanacheewin S, Taweechaisupapong S. Antimicrobial and antibiofilm activity of LL-37 and its truncated variants against *Burkholderia pseudomallei*. Int J Antimicrob Agents. 2012 Jan;39(1):39-44. doi: 10.1016/j.ijantimicag.2011.09.010. Epub 2011 Oct 17. PMID: 22005071.

**8. Why did the MIC values differ from the results presented in Fig 2? If the MIC of AMX against *K. pneumoniae* is at 4.3 uM, why couldn’t it killing the KP in Fig 2 even at 10uM?**

**Response:** With all due respect to the reviewer, the MIC values of AMX against *K. pneumoniae* was presented in Table 3 as 4276 µM or 4.3 mM (1562.5 µg/ml) not 4.3 µM. We have placed comma every three decimals for numbers larger than 999. We thank you for noticing this possible confusion. We have revised the number throughout this manuscript for avoid any confusion.

**9. Fig 3. The AMX and TOB concentrations used were way higher than the clinical MIC breakpoints. Are these concentrations relevant to the CRS treatment?**

**Response:** In this study, we have used the clinical bacterial isolates from CRS patients because of their antibiotic resistance characteristics. Thus, the MIC values of AMX and TOB were higher than MIC breakpoints. Moreover, the IC_50_ values of tested antibiotics were determined against bacteria under biofilm-stimulating condition which enhanced the resistance to antibiotics at least 100-1,000 times compared to their planktonic counterpart.

Although, no antibiotic has been approved by the U.S. Food and Drug Administration for the treatment of CRS. Most experts agree that antimicrobials for treatment of CRS should provide broad-spectrum coverage. Commonly used agents include AMX which is the first line-drug for ABRS (acute bacterial rhinosinusitis). Recommended antibiotic doses for adults with CRS of AMX is 1.5 - 4 g/d, divided BID which was higher than the MIC or IC_50_ values of AMX in this study (ranking from 1.5-16.5 mg/ml).

**10. The failed bacterial culture rate was at 50%. Although a discussion regarding the failed bacterial culture could be resulted from other microbial species (anaerobic bacteria and fungi), it could simply be just the culturing techniques/methods. This is because those microbial species (anaerobic bacteria and fungi) should be rare and existed at much lower percentage as discussed and not likely to be accounted for almost 50% of the failed culture. The authors should either provide additional evidence (PCR or sequencing) or to revise the description.**

**Response:** We thank the reviewer for this constructive suggestion. Until 2012 bacterial culture was the basis of the published studies and the focus was to identify single bacterial species being causative for the disease and a possible target for antimicrobial therapy. Bacterial culture only reveals a small subset of bacteria detectable by culture independent broad-range analysis of 16S rRNA. However, Koeller and colleagues (2018) have examined how the clinically available culture techniques interrelate with the microbiome analyzed via 16S rRNA gene high throughput sequencing of CRS compared to healthy bacterial communities. The results showed that cultivating swabs still served its task to identify a potentially causative pathogen, however 16S rRNA based sequencing analysis revealed higher abundance genera in microbiome analysis than culture techniques. Thus, molecular techniques should be used as addition tool to identify microorganisms in clinical samples. As suggested by the reviewer, we have added the sentence in Discussion section accordingly (page 25, lines 526-529).

“In addition, using standard culture-based methods was the limitation in this study which may affect the exact number of isolated bacteria from CRS patients. Molecular technique should be used as addition tool to identify microorganisms in clinical samples.”

Reference

Koeller K, Herlemann DPR, Schuldt T, Ovari A, Guder E, Podbielski A, Kreikemeyer B, Olzowy B. Microbiome and Culture Based Analysis of Chronic Rhinosinusitis Compared to Healthy Sinus Mucosa. Front Microbiol. 2018 Apr 17;9:643. doi: 10.3389/fmicb.2018.00643. PMID: 29755418; PMCID: PMC5932350.

**11. For Table 5, what were the applicable concentrations of AMX, TOB, and LL-31 used for determining the synergistic action? Are the AMX concentrations used achievable or relevant in clinical conditions? There are a lot of experimental details missing for an effective evaluation of the presented data. In addition, what was the rationale for testing the synergistic effect if your peptide or TOB was already very effective? Why not choose resistant isolates/strains for evaluating the synergistic effect?**

**Response:** Treatment of CRS is aimed at reducing mucosal inflammation, controlling infection, and restoring mucociliary clearance within the sinuses. Topical corticosteroids, saline nasal irrigation, topical antibiotic therapy or sinus surgery are generally used in the medical management of CRS. However, low efficiency of topical antibiotic treatment has been reported according to biofilm-associated sinus infection, which make these infections even more difficult to treat. In this study, we have determined the synergistic interaction of D-LL-31 with conventional antibiotics against bacteria under biofilm condition to enhance biofilm-eradicating effect of currently used antibiotics and to reduce the peptide concentrations to levels high enough to disrupt bacterial biofilms while minimizing potential systemic side effects of topical antibiotic treatment. Recommended antibiotic doses for adults with CRS of AMX is 1.5 - 4 g/d, divided BID which was higher than the MIC or IC_50_ values of AMX in this study (ranking from 1.5-16.5 mg/ml).

Even though D-LL-31 and TOB have a high potency to kill bacteria grown as biofilm (based on IC_50_ values), a combination approach (different mechanism of two antimicrobial agents) has been described to delay of antibiotic resistance rate and reduce the possible side effects of an antibiotic (Grassi et al., 2017). Therefore, combination between D-LL-31 with antibiotics could be used as an effective topical treatment to eliminate and kill the biofilm-embedded bacteria, especially CRS patients.

All three selected bacterial isolates are resistance strain. They exhibited resistance to AMX and also TOB when growing in biofilm-stimulating condition (followed the CLSI 2018 breakpoint) (Tables 3 and 4).

Reference

Grassi L, Maisetta G, Esin S, Batoni G. Combination Strategies to Enhance the Efficacy of Antimicrobial Peptides against Bacterial Biofilms. Front Microbiol. 2017 Dec 7;8:2409. doi: 10.3389/fmicb.2017.02409. PMID: 29375486; PMCID: PMC5770624.

**12. Although the binding data of D-LL-31 to the fixed biofilm matrix is interesting, it could not directly explain if the peptide affect the membrane and disrupt the biofilm especially the nature of positive charged D-LL-31 would be expected to bind the negatively charged bacterial membrane regardless if it disrupts the biofilm matrix.**

**Response:** Thanks reviewer for your important suggestions. Several studies indicated that low penetration of antibiotics to biofilms have been found as major problem of antibiotic resistance (Høiby et al., 2010, Lebeaux et al., 2014). The main biofilm matrix components are polysaccharides, proteins, lipids and extracellular DNA (eDNA) distributed in a non-homogeneous pattern (Flemming and Wingender, 2010), which can contribute the interaction between negatively-charged molecules and positively-charged peptides result in the penetrate and/or to inhibit/interfere the biofilm matrix with its accumulation. In this study, we have performed experiments to show that D-LL-31 was able to rapidly penetrate biofilm architectures and reduce biofilm matrix within 24 h. Moreover, D-LL-31 showed synergistic effects with conventional antibiotics (AMX and TOB) against bacterial embedded in biofilms for all tested isolates. Although, the exact mechanism of AMPs to reduce bacterial biofilm matrix remains unclear, several anti-biofilm mechanisms of AMPs have been proposed such as disruption or degradation of the membrane potential of biofilm embedded cells (Zhu et al., 2013), interruption of bacterial cell signaling systems (De la Fuente-Núñez et al., 2014), degradation of the polysaccharide and biofilm matrix (Beaudoin et al, 2018), inhibition of the alarmone system to avoid the bacterial stringent response (De la Fuente-Núñez et al., 2014) and downregulation of genes responsible for biofilm formation and transportation of binding proteins (Overhage et al., 2008). Our results indicated that perturbation of matrix synthesis and disruption of biofilm architecture caused by AMPs plays a key role in allowing increased access of currently used antibiotics toward biofilm-embedded bacteria.

Reference

Høiby N, Bjarnsholt T, Givskov M, Molin S, Ciofu O. Antibiotic resistance of bacterial biofilms. Int J Antimicrob Agents. 2010 Apr;35(4):322-32. doi: 10.1016/j.ijantimicag.2009.12.011. Epub 2010 Feb 10. PMID: 20149602.

Lebeaux D, Ghigo JM, Beloin C. Biofilm-related infections: bridging the gap between clinical management and fundamental aspects of recalcitrance toward antibiotics. Microbiol Mol Biol Rev. 2014 Sep;78(3):510-43. doi: 10.1128/MMBR.00013-14. PMID: 25184564; PMCID: PMC4187679.

Flemming HC, Wingender J. The biofilm matrix. Nat Rev Microbiol. 2010 Sep;8(9):623-33. doi: 10.1038/nrmicro2415. Epub 2010 Aug 2. PMID: 20676145.

Zhu C, Tan H, Cheng T, Shen H, Shao J, Guo Y, Shi S, Zhang X. Human β-defensin 3 inhibits antibiotic-resistant *Staphylococcus* biofilm formation. J Surg Res. 2013 Jul;183(1):204-13. doi: 10.1016/j.jss.2012.11.048. Epub 2012 Dec 20. PMID: 23273885.

de la Fuente-Núñez C, Korolik V, Bains M, Nguyen U, Breidenstein EB, Horsman S, Lewenza S, Burrows L, Hancock RE. Inhibition of bacterial biofilm formation and swarming motility by a small synthetic cationic peptide. Antimicrob Agents Chemother. 2012 May;56(5):2696-704. doi: 10.1128/AAC.00064-12. Epub 2012 Feb 21. PMID: 22354291; PMCID: PMC3346644.

Beaudoin T, Stone TA, Glibowicka M, Adams C, Yau Y, Ahmadi S, Bear CE, Grasemann H, Waters V, Deber CM. Activity of a novel antimicrobial peptide against *Pseudomonas aeruginosa* biofilms. Sci Rep. 2018 Oct 3;8(1):14728. doi: 10.1038/s41598-018-33016-7. PMID: 30283025; PMCID: PMC6170476.

Overhage J, Campisano A, Bains M, Torfs EC, Rehm BH, Hancock RE. Human host defense peptide LL-37 prevents bacterial biofilm formation. Infect Immun. 2008 Sep;76(9):4176-82. doi: 10.1128/IAI.00318-08. Epub 2008 Jun 30. PMID: 18591225; PMCID: PMC2519444.

**13. Fig 6. A lung cancer cell line A549 was chosen for cell viability studies. A549 is a cancer cell line and it is NOT likely represent the normal nasal epithelial cell response. Can the author justify why A549 is relevant to CRS? Are there any primary nasal epithelial cells available? Also, the epithelial cells were cultured on 96-well plates, which is fine, but there are established air-liquid interface (ALI) cell culture systems for TEER and cell viability studies. The ALI system would be a much better model for the purpose of this study.**

**Response:** We thank the reviewer for this constructive suggestion. Immortal cell lines commonly used to mimic the upper respiratory tract for *in vitro* CRS models are 16HBE (human bronchial epithelial cells), Calu-3 and A549 (cells from human lung carcinoma) (De Rudder et al., 2018). Several CRS studies also used A549 cells as model to investigate the pathogenesis of CRS (Sachse et al., 2006; Shin et al., 2012 and Yang et al., 2017).

ALI conditions allow the cell layer to mimic *in vivo* functionality more closely than immortal cell lines in submerged culture, but require highly specialized and costly apparatus, whereas two-dimensional (2D) submerged culture method has the advantages of easy acquisition, source reliability at low cost. The ability to repeat experiments and yield reproducible results is another benefit (De Rudder et al., 2018).

References

De Rudder C, Calatayud Arroyo M, Lebeer S, Van de Wiele T. Modelling upper respiratory tract diseases: getting grips on host-microbe interactions in chronic rhinosinusitis using in vitro technologies. Microbiome. 2018 Apr 24;6(1):75. doi: 10.1186/s40168-018-0462-z. PMID: 29690931; PMCID: PMC5913889.

Sachse F, von Eiff C, Stoll W, Becker K, Rudack C. Induction of CXC chemokines in A549 airway epithelial cells by trypsin and staphylococcal proteases - a possible route for neutrophilic inflammation in chronic rhinosinusitis. Clin Exp Immunol. 2006 Jun;144(3):534-42. doi: 10.1111/j.1365-2249.2006.03089.x. PMID: 16734624; PMCID: PMC1941973.

Shin HW, Cho K, Kim DW, Han DH, Khalmuratova R, Kim SW, Jeon SY, Min YG, Lee CH, Rhee CS, Park JW. Hypoxia-inducible factor 1 mediates nasal polypogenesis by inducing epithelial-to-mesenchymal transition. Am J Respir Crit Care Med. 2012 May 1;185(9):944-54. doi: 10.1164/rccm.201109-1706OC. Epub 2012 Feb 9. PMID: 22323302.

Yang HW, Lee SA, Shin JM, Park IH, Lee HM. Glucocorticoids ameliorate TGF-β1-mediated epithelial-to-mesenchymal transition of airway epithelium through MAPK and Snail/Slug signaling pathways. Sci Rep. 2017 Jun 14;7(1):3486. doi: 10.1038/s41598-017-02358-z. PMID: 28615628; PMCID: PMC5471256.

**14. Fig 7. It looks like the cells were treated with peptides only? Can the author explain why the cells were not first exposed to bacterial infection and then quantify the cytokines? It’s unlikely that a patient will instill the peptide under normal condition for a prophylactic treatment.**

**Response:** A major problem of CRS patients is an inflammatory immune response and infection (Akdis et al., 2013, Rosenfeld et al., 2015). Thus, treatment of CRS is aimed at reducing mucosal inflammation, controlling infection, and restoring mucociliary clearance within the sinuses. Moreover, parental peptide LL-37 has been reported to associate with immunopathogenesis of CRS (Jiang et al., 2012). Therefore, the immunomodulatory effect of D-LL-31 on A549 cells was performed. Our result showed that no induction of cytokine involving the immunopathogenesis of CRS after treatment of D-LL-31 have been detected. Moreover, a novel role of D-LL-31 in tissue remodeling was observed. The therapeutic application of D-LL-31 may provide benefit to CRS patients by killing antibiotic resistant bacteria, reducing biofilm matrix and promoting tissue-repair of airway extracellular matrix without inflammatory induction.

References

Akdis CA, Bachert C, Cingi C, Dykewicz MS, Hellings PW, Naclerio RM, Schleimer RP, Ledford D. Endotypes and phenotypes of chronic rhinosinusitis: a PRACTALL document of the European Academy of Allergy and Clinical Immunology and the American Academy of Allergy, Asthma & Immunology. J Allergy Clin Immunol. 2013 Jun;131(6):1479-90. doi: 10.1016/j.jaci.2013.02.036. Epub 2013 Apr 12. PMID: 23587334; PMCID: PMC4161279.

Rosenfeld RM, Piccirillo JF, Chandrasekhar SS, Brook I, Ashok Kumar K, Kramper M, Orlandi RR, Palmer JN, Patel ZM, Peters A, Walsh SA, Corrigan MD. Clinical practice guideline (update): adult sinusitis. Otolaryngol Head Neck Surg. 2015 Apr;152(2 Suppl):S1-S39. doi: 10.1177/0194599815572097. PMID: 25832968.

Jiang YY, Xiao W, Zhu MX, Yang ZH, Pan XJ, Zhang Y, Sun CC, Xing Y. The effect of human antibacterial peptide LL-37 in the pathogenesis of chronic obstructive pulmonary disease. Respir Med. 2012 Dec;106(12):1680-9. doi: 10.1016/j.rmed.2012.08.018. Epub 2012 Sep 14. PMID: 22981321.

**15. Line 578 regarding the difference to the LL37. You did not perform the experiment with the available parent peptide LL37 together and this is an overstatement for the results.**

**Response:** We thank the reviewer for this comment. We have deleted the sentence from the discussion section.

**Minor concerns:**

**1. The scale bars of the SEM images were mentioned but not provided, which should be included.**

**Response:** The scale bars were presented in the Fig 1 as dot line. We believe that the line did not clearly enough in the original manuscript. We have revised Fig 1 accordingly.

**2. The statement “These findings indicated that D-LL-31 was more effective than the L-form and the LL-37 variants against all bacteria grown as biofilm” was incorrect as it only showed better activity for *S. epidermidis* strain.**

**Response:** Based on IC_50_ values, D-LL-31 was more effective than the L-form and the LL-37 variants toward all tested isolates (Table 4). Therefore, we have moved this sentence to page 18; lines 391-392 to avoid any misunderstandings.

“However, these findings also indicated that D-LL-31 was more effective than the L-form and the LL-37 variants against all bacteria grown as biofilm.”

**3. Line 517, citation or proof showing that D-LL-31 is protease resistant should be provided.**

**Response:** With all due respect to the reviewer, we had already mentioned this point in the manuscript in Introduction section (Page 4, lines 76-78).

“The substitution of L- by D-amino acids in these peptides improves their stability, enhances their antimicrobial activity and reduces toxicity [20].”

Moreover, the proteolytic resistance of D-enantiomer cathelicidin LL-37 peptide has been reported. Dean and colleagues found that the protease trypsin does not degrade D-LL-37 while L-LL-37 is completely degraded and therefore experiences a loss of function.

Reference

Dean SN, Bishop BM, Van Hoek ML. 2011. Susceptibility of *Pseudomonas aeruginosa* biofilm to alpha-helical peptides: D-enantiomer of LL-37. Front Microbiol 2:128. https://doi.org/10.3389/fmicb.2011.00128 PMID: PMC3131519

**4. A better proofreading should be performed. For instance, line 488, the “enhancement” biofilm-eradicating effect of…..**

**Response:** Thank you for careful reading of our manuscript. As suggested by the reviewer, we have proofread the manuscript and revised it accordingly (For line 488, see page 24; line 508).

“In this study, the enhancement of biofilm-eradicating effect of currently used antibiotics for CRS treatment with cathelicidin-derived peptides and D-enantiomers were observed.”

**5. In line 553, what does it mean for “Indeed, D-LL-31 alone could reduce biofilm matrix to a greater extent than did either antibiotic.” It did not seem to be in accordance with the presented data of TOB.**

**Response:** With all due respect to the reviewer, this sentence referred to the result of biofilm matrix reduction (Fig 4) not the killing activity (Fig 3). Although the killing activity of TOB against *P. aeruginosa* biofilm was comparable to D-LL-31 (Table 4; IC_50_), D-LL-31 displayed significantly (*P* >0.01) greater biofilm-reduction properties than TOB (Fig 3C).

We consider that the manuscript has improved significantly as a result of the comments. We hope that you find our responses satisfactory and that the manuscript is now acceptable for publication in PlosOne.

We look forward to hearing from you soon.

Respectfully yours,

Asst.Prof.Dr. Sakawrat Kanthawong

Department of Microbiology, Faculty of Medicine,

Khon Kaen University, Khon Kaen, Thailand 40002

E-mail: sakawrat@kku.ac.th

Telephone number: 66-89-8483-404
